# An epigenetic vaccine model active in the prevention and treatment of melanoma

**DOI:** 10.1186/1479-5876-5-64

**Published:** 2007-12-10

**Authors:** A Nazmul H Khan, William J Magner, Thomas B Tomasi

**Affiliations:** 1 Laboratory of Molecular Medicine, Department of Immunology, Roswell Park Cancer Institute, Buffalo, NY, USA; 2 Departments of Medicine and Microbiology & Immunology, School of Medicine and Biomedical Sciences, State University of New York, Buffalo, NY, USA

## Abstract

**Background:**

Numerous immune genes are epigenetically silenced in tumor cells and agents such as histone deacetylase inhibitors (HDACi), which reverse these effects, could potentially be used to develop therapeutic vaccines. The conversion of cancer cells to antigen presenting cells (APCs) by HDACi treatment could potentially provide an additional pathway, together with cross-presentation of tumor antigens by host APCs, to establish tumor immunity.

**Methods:**

HDACi-treated B16 melanoma cells were used in a murine vaccine model, lymphocyte subset depletion, ELISpot and Cytotoxicity assays were employed to evaluate immunity. Antigen presentation assays, vaccination with isolated apoptotic preparations and tumorigenesis in MHC-deficient mice and radiation chimeras were performed to elucidate the mechanisms of vaccine-induced immunity.

**Results:**

HDACi treatment enhanced the expression of MHC class II, CD40 and B7-1/2 on B16 cells and vaccination with HDACi-treated melanoma cells elicited tumor specific immunity in both prevention and treatment models. Cytotoxic and IFN-γ-producing cells were identified in splenocytes and CD4^+^, CD8^+ ^T cells and NK cells were all involved in the induction of immunity. Apoptotic cells derived from HDACi treatments, but not H_2_O_2_, significantly enhanced the effectiveness of the vaccine. HDACi-treated B16 cells become APCs in vitro and studies in chimeras defective in cross presentation demonstrate direct presentation in vivo and short-term but not memory responses and long-term immunity.

**Conclusion:**

The efficacy of this vaccine derives mainly from cross-presentation which is enhanced by HDACi-induced apoptosis. Additionally, epigenetic activation of immune genes may contribute to direct antigen presentation by tumor cells. Epigenetically altered cancer cells should be further explored as a vaccine strategy.

## Background

Modified tumor cells can induce tumor-specific immunity and, in certain models, activate both adaptive and innate immune responses [1]. However, in some mouse models and the vast majority of human cancers, the tumor vaccines currently employed have not been successful. This may be attributed to a failure of adequate stimulation of appropriate components of immunity and/or tolerance to tumor antigens [2]. In general, tumor vaccination strategies have focused on enhancing a cytotoxic T cell (CTL) response. Activation of both T-helper cells and CTLs is achieved primarily through cross-presentation of tumor antigens by professional antigen presenting cells (APCs) [2]. Antigens from apoptotic cells have been reported to be a preferred vehicle for activating tumor immunity, rather than tolerance, through cross-presentation by APCs [1,3]. Direct antigen presentation by tumor cells could potentially activate T cells provided the tumor cells can deliver an MHC-restricted antigen-specific signal together with appropriate costimulatory signals [4-6]. However, the role of tumor cells as APCs has not been well defined. Nevertheless, MHC class I mediated direct priming of CTLs has been observed in an engineered tumor model which is dependent on the density of MHC/peptide complexes and the expression of B7 costimulatory molecules on tumor cells [7]. Moreover, transfection of MHC class II negative tumors with MHC class II and B7-1 genes produces a cellular vaccine capable of eliciting immunity [8]. MHC class II positive tumor cells are also effective APCs in vivo and can present novel endogenous antigenic peptides not presented by host APCs [5]. Furthermore, transfection of tumors with class II transactivator (CIITA) elicits MHC class II expression and can restore the ability of certain tumor cells to present antigen and induce immunity [9,10]. Although cross-presentation is the major mechanism generating immunity [2,3], the above studies on tumors as APC suggest that, at least in certain tumors, direct antigen presentation could provide an alternative or additional pathway in tumor immunity. An important issue is whether direct presentation can be enhanced in vivo and become a quantitatively significant component of tumor immunity.

Tumor escape has been attributed to selection of tumor cells with mutations in genes involved in both the initiation and effector phases of immunity [11]. Recent evidence also suggests that tumor cells may exploit epigenetic silencing of immune genes to escape immune destruction [12]. Epigenetic repression of immune genes in tumors was first suggested for MHC class II genes and CD40, which are infrequently mutated although often deficient in tumors [13]. A number of other immune genes, including MHC class I, components of the class I peptide presentation pathway (TAP1, TAP2, LMP2, LMP7 and Tapasin), B7-1/2, NKG2D ligands and certain tumor antigens, are also silenced by chromatin in multiple tumor types [12,14].

Covalent modifications of chromatin are well established regulators of gene expression and an array of epigenetic alterations, including acetylation and methylation, have been shown to target histones [15]. Histone acetylation induced by histone deacetylase inhibitors (HDACi) frequently increases accessibility of transcription factors to promoter sites and results in enhanced gene transcription, although some genes are inhibited by HDACi. The HDACi trichostatin A (TSA) regulates the expression of ~5% of the genome [16]. HDACi treatments have been used in clinical trials on the basis of their ability to induce differentiation and apoptosis of tumor cells [17]. HDACi-treated tumor cells have also been successfully employed in a murine tumor vaccine model [4]. Reversal of gene silencing by HDACi treatment can convert a plasma cell tumor to an APC, in vitro, through upregulation of MHC class II and costimulatory molecules [4,18]. Moreover, HDACi treated melanoma tumor cells can mediate direct antigen presentation via MHC class I, which stimulates IFN-γ-producing T cells in vitro [14]. The present study was constructed to determine the mechanisms involved in immunity generation by TSA-treated melanoma vaccines and whether this epigenetically modified tumor cell vaccine is effective in a treatment model.

## Materials and methods

### Cells, mice and reagents

Mouse B16 melanoma, EL4 thymoma cell lines and hybridoma GK1.5, 2.43 and 53-6.7 cells (ATCC, Manassas, VA) were maintained in culture as specified. Six to eight week old female C57BL/6 (B6) (NCI, Bethesda, MD), MHC class I deficient B6.129-β_2_m^tm1 ^[*class I*^-/-^], class II deficient B6.129-H2^dlAb1-Ea ^(*class II*^-/-^) (Jackson Laboratory, Bar Harbor, ME), class I and class II double deficient B6.129-Aββ^tm1^-β_2_m^tm1 ^(*class I*^-/-^*-II*^-/-^) (Taconic, Germantown, NY) and ovalbumin-specific I-A^b^-restricted TCR transgenic (OT-II) (Protul Shrikant, RPCI, Buffalo, NY) mice were maintained in the Department of Laboratory Animal Resources at RPCI. Principles of laboratory animal care (NIH publication 85-23, revised 1986) were followed and all work was carried out under RPCI IACUC approval. TSA (Wako Biochemical, Richmond, VA), H_2_O_2 _(Sigma, St. Louis, MO) and mouse IL-2 (R&D System, Minneapolis, MN) were diluted in ethanol, water and PBS containing 0.5% BSA, respectively.

### Flow cytometry

R-Phycoerythrin conjugated anti-mouse I-A^b^, H-2D^b^, CD4, CD40, CD80, CD86 and Pan-NK (DX5), FITC conjugated anti-mouse I-A^b ^and CD3, PE-Cy5.5 conjugated anti-mouse CD8 and CD11c mAb, isotype controls matched to each antibody (Pharmingen, San Diego, CA) and FITC conjugated annexinV (Caltag, Burlingame, CA) were used in flow cytometry experiments as previously described [4]. The gated cell populations included both apoptotic and non-apoptotic cells. In all preparations utilized here, the adherent cells studied were >95% viable by trypan blue exclusion.

### Tumorigenesis assays

TSA-treated or untreated B16 cells (1 × 10^5 ^trypan blue negative adherent cells) were injected s.c. in the ventral trunk of the mice. This tumor challenge dose is 10 fold higher than the minimum number of untreated cells required to generate palpable tumor in 100% of mice within 3 weeks after injection. Treated cells were assayed prior to injection to ensure consistency between experiments in MHC and costimulatory molecule expression as well as apoptosis. Tumors were measured every 3 days and mice were euthanized when tumor diameter reached 1 cm. After 40 days, all tumor-free mice were re-challenged s.c. with untreated B16 (1 × 10^5^) cells in the opposite site and observed for another 60 days. In addition to palpable tumor measurement, the absence of evidence of tumor in immune mice was confirmed by visual inspection. At the end of the study period (100 days after vaccination), a mouse from each group of tumor-free mice was euthanized and the tumor inoculation sites and regional lymph nodes dissected and examined for evidence of tumor (no visible tumor nodule or other evidence of tissue aberration) and compared with a palpable tumor-bearing mouse (visible tumor nodules in lymph nodes). Some tumor-free B6 mice were re-challenged with EL4 (1 × 10^4^) cells simultaneously as a control for tumor specificity. For the treatment model, B6 mice bearing palpable (~0.5 mm) B16 tumor, 5 days after s.c implantation in the trunk, were vaccinated with TSA- treated and irradiated (2000 Gy) B16 cells in the opposite side. Groups of tumor-bearing control mice received irradiated B16 cells or were left untreated. Mice that became tumor-free were re-challenged 42 days latter with wild type B16 in the trunk and observed for another 40 days.

### Cytotoxicity assays

To determine tumor immunity, tumor-free mice, 30 days after vaccination with TSA-treated B16 cells, were re-challenged with untreated B16 cells and observed for an additional 15 days. Spleens isolated from three immune or control mice were disrupted and splenocytes were purified over nylon wool (Polyscience, Warrington, PA). Non-adherent lymphocytes (5 × 10^5^) were re-stimulated with TSA-treated (500 nM for 48 h) and irradiated (200 Gy) tumor cells (2.5 × 10^5^) in RPMI-1640 with IL-2 (10 U/ml). After 4 days, T-cell-enriched (>90%) viable cells were isolated after centrifugation through Ficoll-Paque and the level of anti-B16 cytotoxicity was determined utilizing a standard 4.5 h ^51^Cr-release assay [4].

### ELISpot assays

The mouse IFN-γ ELISpot kit (BD Bioscience, San Diego, CA) was used to determine antigen specific IFN-γ-secreting cells in spleens of immune mice as described [19]. Briefly, RBC-depleted splenocytes (1 × 10^6 ^cells/well) isolated from immune and control mice were incubated in triplicate wells with B16 tumor cell lysate (~8 × 10^4 ^cell lysate/well) or 5 μM mgp100_25–33 _peptide (Invitrogen, Grand Island, NY) for 24 h. IFN-γ spots were developed with AEC substrate and counted using a Zeiss Imaging system. B6 mice bearing palpable tumor 15 days after inoculation of untreated B16 (1 × 10^5 ^cells) and naïve mice were used as controls. Splenocytes (2 × 10^5 ^cells/well) from naïve mice treated with PMA (40 ng/ml) and Ionomycin (1 μM) served as positive control. For in vitro antigen presentation assays, popliteal lymph node T cells (2 × 10^5^), isolated from ovalbumin-primed OT-II mice and purified magnetically (Pan T cell isolation kit, Miltenyi, Auburn, CA), were assayed in triplicate with TSA-treated (500 nM for 48 h) or untreated B16 cells (1 × 10^5^) for 24 h. Splenocytes isolated from OT-II mice were used as control APCs. Tumor cells and splenocytes were pulsed with ova-peptide_322–338 _or the control E333A ova-peptide_322–338 _(10 μM) and irradiated (2000 Gy and 30 Gy respectively) before use in the ELISpot assays to measure IFN-γ-secreting T cells.

### *In vivo *depletion studies

Anti-CD4 (GK1.5) and CD8 (2.43) hybridomas were grown i.p. in SCID mice and ascites fluid was partially purified by ammonium sulfate precipitation. To deplete CD4^+ ^or CD8^+ ^T cells, B6 mice were injected i.p. with CD4 or CD8 mAb (200 μg in 200 μl PBS/mouse), given at day -2, 0, +2, +4, +8 and +14. Rabbit anti-asialoGM1 γ-globulin (Wako), reconstituted in water and diluted in PBS, was used for NK cell depletion using the same schedule. Control mice received purified rat IgG (Chemicon International, Temecula, CA). Control and depleted mice were vaccinated with TSA-treated B16 cells after the 2^nd ^dose of antibody injection and observed for 30 days. Depletion was assessed on the day of vaccination and a week later by flow cytometric analysis of splenocytes for CD3^+^CD4^+^, CD3^+^CD8^+ ^T cells and DX5^+ ^NK cells.

### Bone marrow chimera generation

Bone marrow (BM) cells were harvested from femur and tibia of groups of donor (B6 and *class I*^-/-^*-II*^-/-^) mice by flushing the bones with RPMI-1640. RBCs were lysed using Tris-buffered ammonium chloride and single cell suspensions were obtained by passing BM cells through a cell strainer [20]. T cells were depleted from BM preparations by incubation with CD8 (53-6.7) and CD4 (GK 1.5) mAb (1:10 dilution) for 60 min at 4°C, followed by lysis with low-tox-M rabbit complement (Cederlane, Ontario, Canada) for 90 min at 37°C in 5% CO_2 _incubator [21]. In preliminary experiments, BM cells after treatment with hybridoma supernatants (53-6.7 or GK1.5) were analyzed by flow cytometry using CD3, CD4 (L3T4) and CD8 (5H10) mAb to confirm the depletion of CD3^+^CD4^+ ^or CD3^+^CD8^+ ^T cells. One day before BM infusion, recipient B6 mice were injected i.p. with a single dose anti-asialoGM1 (20 μl in 200 μl sterile PBS) antibody to prevent NK mediated rejection of BM. The recipient mice were exposed to 11 Gy total body irradiation (TBI) administered in two treatments 3 h apart from a ^137^Cs-radiation source on the day of BM infusion. A total of 5 × 10^6 ^BM cells from donor mice were injected into the tail vein of each recipient. Chimeras were provided with water containing 2 mg/ml neomycin sulfate for 4 weeks after irradiation. Splenocytes, isolated from irradiated mice 2 days after TBI, were analyzed by flow cytometry to confirm the elimination of CD11c^+ ^APCs and CD3^+ ^T cells and >99% elimination was observed. To obtain a dendritic cell (DC) enriched APC population, low-density splenocytes were isolated by collagenase digestion [22]. At 4–8 weeks after BM transplant, chimerism was evaluated using PBLs and splenocytes isolated from chimeric mice.

### Isolation of apoptotic tumor cells

Apoptotic B16 cells were isolated using annexinV microbead kit, MS separation column and magnetic separator (Miltenyi, CA) according to the manufacturer's protocol. Briefly, TSA or H_2_O_2_-treated B16 cells were labeled with annexinV microbead for 15 min at 6–12°C and passed through a separation column in a magnetic field. AnnexinV positive (an^+^) apoptotic cells were retained in the column while annexinV negative (an^-^) non-apoptotic cells ran through. Apoptotic cells were eluted after removal of the column from the magnetic field.

## Results

### Enhanced expression of MHC class II and costimulatory molecules on melanoma cells after TSA treatment

**Figure 1:**
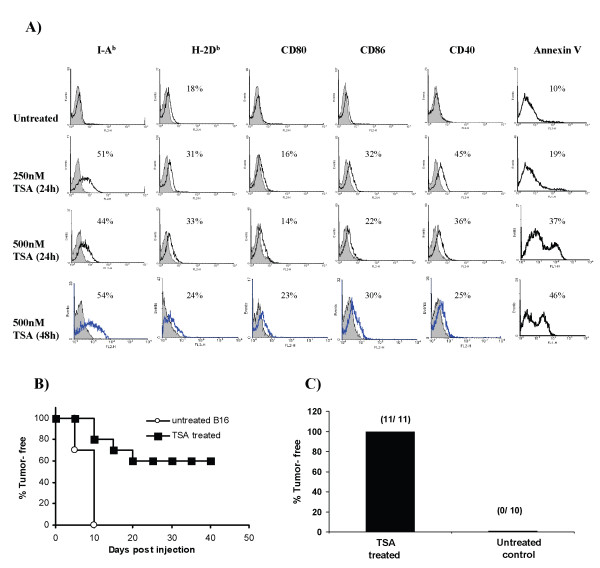
TSA treatment enhances apoptosis and immune gene expression on melanoma cells and TSA-treated vaccines generate immunity. **A**) B16 cells were stained with mAb, isotype controls and annexinV after treatment with TSA and analyzed by flow cytometry for the expression of MHC class I, class II, CD80, CD86 and CD40. Isotype controls are shown as shaded peaks and heavy lines represent expression determined by specific mAb staining. Values indicated in the histograms are the percent of cells positive for the respective mAb relative to the isotype staining. The data presented here are representative of more than three independent experiments. **B**) Kaplan-Meier plot of B6 mice (10 in each group) inoculated with TSA-treated (500 nM for 48 h) or untreated B16 cells in the trunk. **C**) Durable immunity in 100% of the immune animals. Tumor-free mice, 40 days after vaccination with TSA-treated B16, were re-challenged with untreated B16 cells and observed for another 60 days. The number of tumor-free mice compared to total numbers used in each treatment group is shown in parentheses.

Previously we showed that TSA treatment elicits the expression of MHC class II and costimulatory molecules on the mouse plasma cell tumor J558 and that vaccination with epigenetically altered tumor cells generates tumor-specific immunity [4]. To extend this to another tumor and to further analyze the mechanisms involved in immunity generation we used the MHC class II negative B16 melanoma. To determine the cell surface expression of MHC class I, class II, CD40, CD80 and CD86 in relation to apoptosis induction, TSA-treated (50 nM-1 μM, 12–48 h) adherent (~95% viable) B16 cells were analyzed by flow cytometry. The conditions inducing maximal expression are presented in Figure 1A. This Figure demonstrates enhancement of the expression of MHC class II, CD40 and CD86 on B16 cells at 24 h and 48 h of TSA treatment. TSA treatment also slightly enhanced the low-level expression of class I found on untreated cells. Treatment with 500 nM TSA for 48 h, a condition that produced 46% an^+ ^cells in the adherent B16 population, in addition to class II, CD40 and CD86 induced low-level expression of CD80. TSA-treated B16 cells retained enhanced expression of these genes over 24 h after withdrawal of treatment in vitro (data not shown). These results demonstrate that TSA treatment induces apoptosis and elicits expression of class II and costimulatory molecules on B16 tumor cells similar to the results previously reported on the J558 plasmacytoma [4].

### Durable immunity generated by epigenetically altered melanoma cell vaccination

To determine whether TSA treatment altered tumorigenicity and immunogenicity, B16 cells treated with different concentrations of TSA were inoculated into mice. As shown in Table 1, nearly all control and 90% of the 250 nM TSA treated B16 (~20% an^+ ^cells) injected mice developed tumors. Using 500 nM TSA (24 h) treated B16 (~40% an^+ ^cells), 30% of the mice injected were tumor-free for more than 40 days. However, when mice were inoculated with B16 cells treated with 500 nM TSA for 48 h (~50% an^+ ^cells), 80% of the mice were tumor-free after 10 days, by which time all controls had developed tumors, and more than 60% of the mice remained tumor-free for over 40 days (Table 1 and Figure 1B). When tumor-free immune mice were re-challenged with untreated B16, 100% of the TSA-treated B16 vaccinated mice showed lasting immunity (Figure 1C). The immunity generated by TSA-treated B16 vaccination is tumor specific as the unrelated EL4 thymoma developed tumor in 100% of the immune mice (data not shown). In similar experiments, B16 cells treated with high dose (750 nM for 48 h) TSA, containing ~90% an^+ ^cells, demonstrated immunity in 40% of the mice after vaccination (data not shown). This high dose was associated with greater number of an^+ ^cells did not improve the effectiveness of the vaccine. These results indicate that, similar to the TSA-treated plasmacytoma vaccine, TSA-treated melanoma cells generate immunity and preparations containing approximately 50% an^+ ^cells were most effective in inducing tumor specific lasting immunity [4].

**Table 1:** Tumorigenesis in the epigenetic melanoma vaccine model

Treatment^a^	% AnnexinV^+ ^cells/inocula	Number of mice that developed tumor (total)^b^	% Tumor-free mice after 40 days
Untreated	~10	32 (33)	3
250 nM TSA (24 h)	~20	9 (10)	10
500 nM TSA (24 h)	~40	7 (10)	30
500 nM TSA (48 h)	~50	19 (48)	61

^a ^TSA-treated (concentrations and length of treatment as indicated) or untreated B16 cells (1 × 10^5 ^trypan blue negative adherent cells) were inoculated s.c. in B6 mice. ^b ^The total number of mice used per treatment group is shown in parentheses.

### Induction of cytotoxic and IFN-γ secreting lymphocytes in immune mice

**Figure 2:**
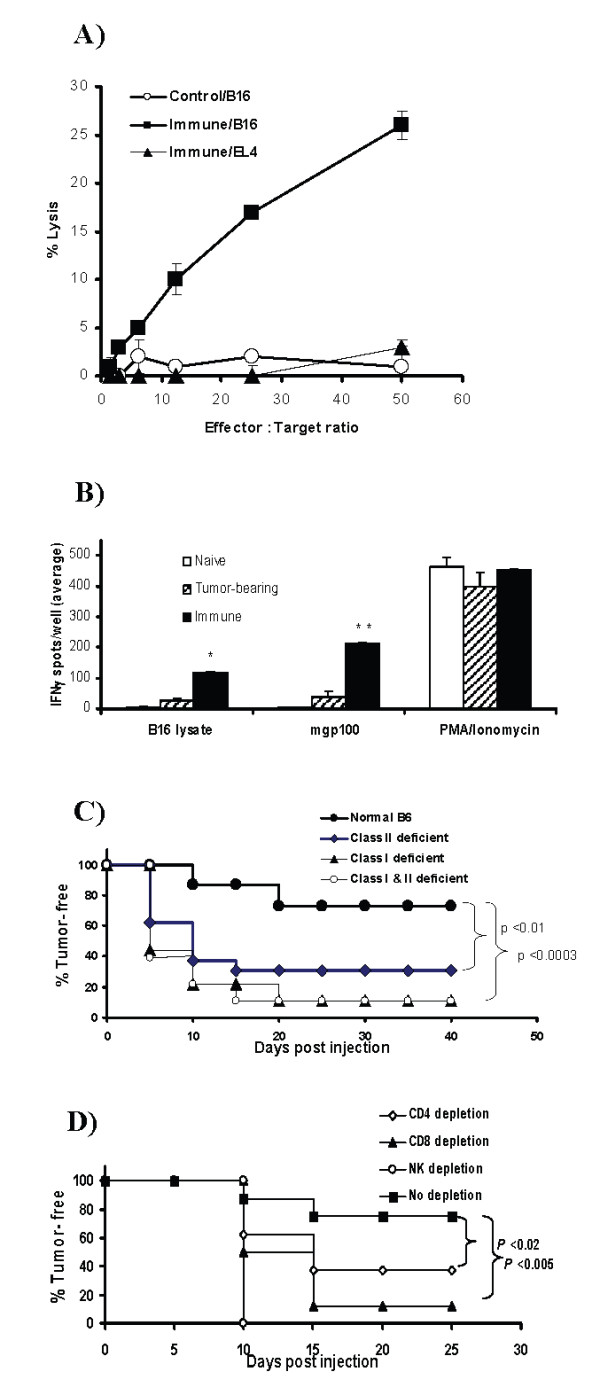
TSA-treated B16 vaccine elicits cytotoxic and IFN-γ-producing lymphocytes and requires T and NK cells in immunity. **A**) T-cell-enriched splenic lymphocytes isolated from immune or control mice after in vitro re-stimulation were analyzed in triplicate for cytotoxic activity using ^51^Cr-labeled untreated B16 or EL4 as targets. **B**) Splenocytes isolated from immune, B16 tumor-bearing or naive B6 mice were cultured in anti-IFN-γ coated plates with B16 cell lysate or melanoma antigen mgp100_25–33 _peptide and IFN-γ secretion was detected by ELISpot assay. *, *p *<0.0008 (cell lysate stimulation) and **, *p *<0.03 (peptide stimulation) compared with splenocytes from tumor-bearing mice. PMA and Ionomycin treated splenocytes served as positive control. These experiments (A and B) were repeated 3 times with similar results. **C**) Reduced incidence of TSA-treated (500 nM for 48 h) B16 tumor rejection in MHC deficient mice that lack CD4^+ ^and/or CD8^+ ^T cells compared with immunocompetent mice. The Kaplan-Meier plot shows tumor-free survival of *class II*^-/- ^(n = 16), *class I*^-/- ^(n = 18), *class I*^-/-^*-II*^-/- ^(n = 18) and immunocompetent B6 (n = 15) mice after s.c. vaccination with TSA-treated B16 (1 × 10^5 ^cells). **D**) Depletion of CD4^+^, CD8^+ ^T cells and NK cells reduces immunity generation by TSA-treated B16 cells. B6 mice were depleted of CD4^+ ^(n = 16), CD8^+ ^(n = 8) T cells or NK cells (n = 8) by repeated injection of respective antibodies (see Methods). Undepleted controls (n = 16) received IgG. All mice were observed for tumor growth after s.c. vaccination with TSA-treated B16 cells in the trunk.

To determine whether CTLs were elicited in immune mice, T-cell-enriched splenic lymphocytes were isolated from TSA-treated B16-vaccinated mice that remained tumor-free after re-challenge and assessed for their ability to lyse untreated B16 cells. As shown in Figure 2A, splenic lymphocytes derived from immune mice displayed substantial B16 lytic activity (26%) compared with splenocytes derived from control mice (<2%) at an effector: target ratio 50:1. The cytotoxic lymphocytes induced by TSA-treated melanoma vaccination were tumor specific, since lysis of the unrelated target EL4 (1%) was significantly lower than that of B16 (Figure 2A).

ELISPOT assays were employed to measure IFN-γ production by cytotoxic lymphocytes from immune mice. As shown in Figure 2B, splenocytes derived from mice immunized with TSA-treated B16 and challenged with untreated tumor cells showed significant increases in the numbers of IFN-γ-producing cells after B16 cell lysate or mgp100_25–33 _peptide stimulation compared to splenocytes derived from naive mice. The number of IFN-γ-producing cells found in splenocytes isolated from B16 tumor-bearing mice was significantly lower than the immune mice. PMA/Ionomycin treatment induced a high number of IFN-γ-producing cells in splenocytes isolated from naïve, tumor-bearing and immune mice. The presence of cytotoxic and IFN-γ-producing cells in the spleens of immune mice, together with the demonstration of long-term immunity after vaccination with TSA-treated B16 cells, suggest that epigenetically altered tumor cells are capable of inducing cell mediated tumor specific effectors and immunity.

### Involvement of CD4^+^, CD8^+ ^T cells and NK cells in immunity induced by epigenetic vaccine

To determine the role of CD8^+ ^and CD4^+ ^T cells in immunity, MHC double knockout *class I*^-/-^*-II*^-/- ^and single knockout *class I*^-/- ^or *class II*^-/-^mice were used in tumorigenesis experiments, since these MHC disrupted mice lack the related subset of mature T cells (data not shown) [23]. The incidence of tumor rejection in *class I*^-/-^*-II*^-/-^, *class I*^-/- ^and *class II*^-/- ^mice (Figure 2C-11%, 11%, 31% tumor-free respectively) 40 days after TSA-treated B16 inoculation, is significantly lower than in immunocompetent B6 mice (73% tumor-free). Although, the percentage of tumor-free mice in the *class II*^-/- ^group is numerically higher than the *class I*^-/- ^group, the difference was not statistically significant (p = 0.08). In subsequent re-challenge with untreated B16, all the MHC deficient mice developed tumors (data not shown) while immunocompetent mice remained tumor-free.

To more specifically evaluate the contribution of T cell subsets to protective immunity generated by TSA-treated tumor cell vaccination, in vivo depletion was performed. Calculation of the total number of CD4^+ ^and CD8^+ ^T cells in each spleen, isolated from the respective T cell depleted mice, compared to undepleted control spleens, demonstrated >95% depletion of specific subsets (data not shown). Figure 2D shows that the percentage of tumor-free mice in CD8^+ ^or CD4^+ ^T cell depleted groups (12.5% and 37.5%, respectively), 3 weeks after vaccination with TSA-treated B16 cells, is significantly lower than the control groups (~75%). NK cells can be responsible for tumor rejection by direct lysis of tumor cells and by producing cytokines that recruit and activate DCs and T cells [24,25]. Depletion of NK cells prior to vaccination, as shown in Figure 2D, completely abrogated the anti-tumor effect of TSA-treated B16 vaccination. These results demonstrate that CD4^+^, CD8^+ ^T cells and NK cells are all required for generation of the immune response by TSA-treated B16 cells and that NK cells are particularly important in tumor rejection. However, the TSA-treated B16 tumorigenesis data from T cell deficient *class I*^-/-^*-II*^-/- ^mice and our previous studies in SCID mice with TSA-treated plasmacytoma [4] suggest that NK cells alone, in the absence of T cells, are not sufficient to induce effective anti-tumor immunity in these epigenetic vaccine models.

### Antigen presentation by TSA treated tumor cells *in vitro *and *in vivo*

**Figure 3:**
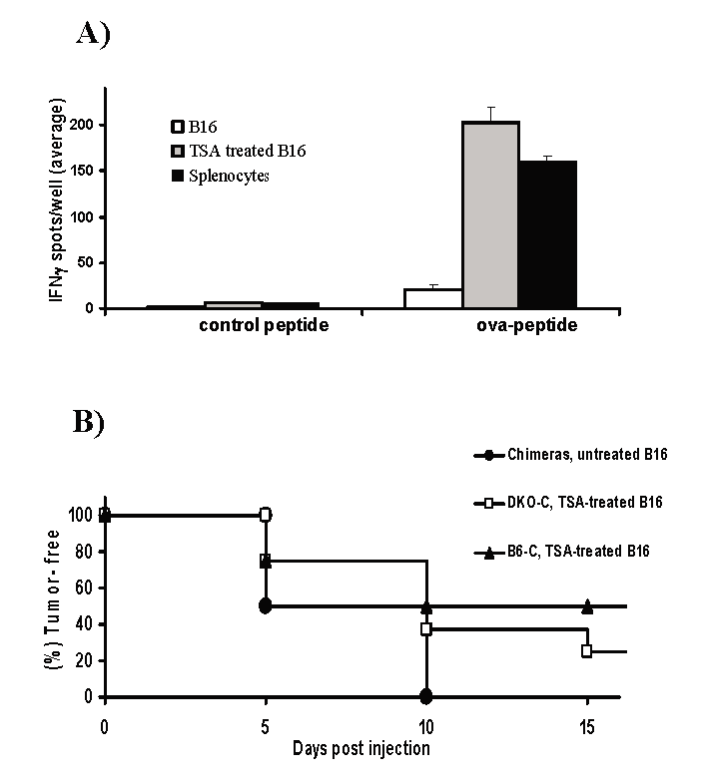
TSA treatment converts B16 melanoma cells to APCs. **A) **In vitro antigen presentation by TSA-treated (500 nM for 48 h) B16 cells. Purified T cells from OT-II mice were cultured with antigen-pulsed (ova-peptide_322–338 _or control peptide), irradiated B16 cells (TSA-treated or untreated) and splenocytes (from OT-II mice as control APCs) in a standard ELISpot assay to measure IFN-γ-secreting cells. **B**) Eight weeks after BM transplantation, control [B6-C] and MHC double knockout [DKO-C] chimeras were inoculated s.c. with TSA-treated B16 (1 × 10^5 ^cells) in the trunk and tumor-free survival was recorded. Control groups of B6-C and DKO-C chimeras were injected with untreated B16 cells. The survival curve for these two groups were coincident; therefore a representative plot [chimeras, untreated B16] showing the tumor-free survival of these controls is presented. Results from two independent experiments (four chimeras/group/experiment) are presented in this figure.

In order to determine the mechanism of epigenetic vaccine induced immunity, we further explored whether B16 melanoma cells are converted to APC following TSA treatment. Figure 3A demonstrates that TSA-treated B16 cells can present ova-peptide_322–338 _to OT-II T cells in the context of MHC class II in vitro. In view of these studies and to determine the role of direct antigen presentation in elicitation of the immune response by epigenetic tumor cell vaccination in vivo, we developed MHC double knockout BM (DKO-C) chimeras using *class I*^-/-^*-II*^-/- ^donors and irradiated B6 recipients. Control BM (B6-C) chimeras were produced using immunocompetent B6 donors and irradiated B6 recipients. Both class I and class II expressing APCs were significantly reduced in DKO-C chimeras and they were repopulated with CD4^+ ^and CD8^+ ^T cells (see Additional file 1). Since MHC class I and/or class II mediated antigen presentation by BM derived APCs initiates cross priming of T cells [1], the DKO-C chimeras are expected to have lost the ability to present antigen from cross priming. B6-C chimeras did not show a deficiency in APC MHC expression, were re-populated with T cells (see Additional file 1) and were expected to have intact cross-presentation capability from both MHC pathways. When these chimeras were inoculated with TSA-treated or untreated B16 cells, 50% of control chimeras and 37.5% of DKO-C chimeras remained tumor-free 10 days after inoculation of TSA-treated B16 cells while all the B6-C and DKO-C chimeras had developed untreated B16 tumors (Figure 3B). Delayed tumor generation, at day 10, in B6-C (p <0.01) or DKO-C chimeras (p <0.03) after inoculation of TSA-treated cells was statistically significant compared to B6-C or DKO-C chimeras injected with untreated B16. These data demonstrate that, in the absence of cross-presentation, tumor generation is delayed suggesting that direct antigen presentation by TSA-treated tumor cells may contribute to delayed tumor growth. However, neither control nor MHC deficient chimeras were able to provide the long-term protection after vaccination with TSA-treated B16 cells that was demonstrated by the immunocompetent mice (Figure 1). This observation also suggests that the BM chimeras used for these experiments, although repopulated with T cells, may not be completely immunocompetent as has been noted by others [26]. Additionally, a recent report suggests that direct presentation by TSA-treated B16 cells may generate partial activation of T cell effector function, but is unable to provide long-term protection in the absence of other signals [27]. This may, at least in part, explain the ability in our chimeric experiments to elicit short-term but not long-term immunity.

### Role of apoptotic tumor cells in the generation of immunity

**Figure 4:**
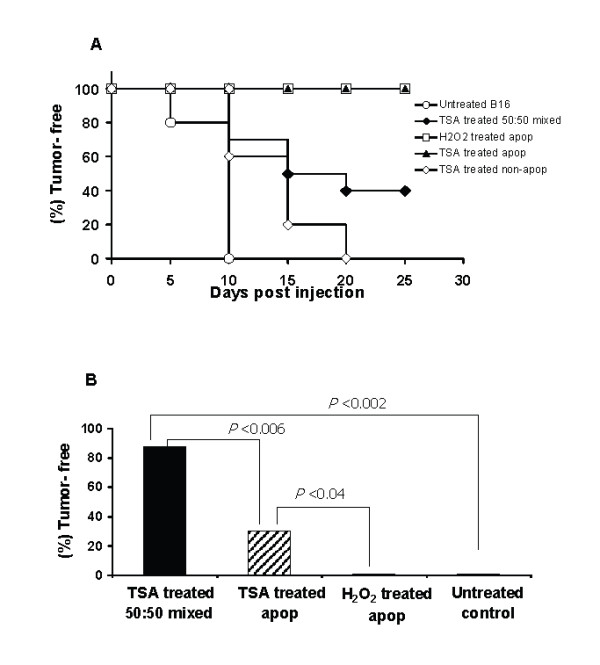
Anti-tumor immunity generated by TSA-treated apoptotic B16 cells. **A**) Kaplan-Meier plot of B6 mice (10 mice/group) vaccinated s.c. with magnetically separated TSA-treated (500 nM for 48 h) apoptotic (~99% an^+^), non-apoptotic (>85% an^-^), mixed (50% an^+ ^with 50% an^-^) or H_2_O_2_-treated (10 mM for 4 h) apoptotic (~99% an^+^) B16 (1 × 10^5 ^cells) and observed for tumor-free survival. Control mice received untreated B16 cells. **B**) Tumor-free mice, 3 weeks after vaccination with TSA-treated apoptotic (n = 10), non-apoptotic (n = 8), mixed (n = 8) or H_2_O_2_-treated apoptotic (n = 10) B16 cells were re-challenged with untreated B16 (1 × 10^5 ^cells) s.c. in the opposite side of the trunk and observed subsequently for another 6 weeks. The data are presented as the percentage of tumor-free mice after tumor challenge.

The tumorigenesis studies with B16 cells (Table 1) showed that the highest level of immunity was obtained using vaccine preparations containing apoptotic cells. This raises the possibility that TSA treatment enhances immunity, at least in part, by induction of apoptosis. To further elucidate the role of apoptotic cells in the B16 model and the ratio of apoptotic to viable cells required for optimal immunity, populations of apoptotic and non-apoptotic adherent B16 cells were separated using annexinV magnetic beads. We also compared the effects of TSA-apoptotic cells with those elicited by H_2_O_2_, a potent inducer of apoptosis via the oxidative pathway [28]. The dose of H_2_O_2 _was determined by titration of B16 cells with H_2_O_2 _at various concentrations and incubation times with apoptosis measured by flow cytometry. Flow cytometric analysis of the separated TSA or H_2_O_2_-treated B16 populations showed that >99% of the cells in the apoptotic fraction were an^+ ^and >85% cells in the non-apoptotic fraction were an^- ^(data not shown). To determine tumorigenicity and immunity, groups of mice were inoculated with TSA or H_2_O_2_-treated apoptotic cells, TSA-treated non-apoptotic or mixed (50% TSA-treated apoptotic + 50% TSA-treated non-apoptotic) B16 cells. As shown in Figure 4A, 40% of mice that received mixed B16 inocula were tumor-free at day 20, while all the mice injected with TSA-treated non-apoptotic or untreated B16 cells developed tumors. TSA or H_2_O_2_-treated apoptotic cells did not replicate in vitro (data not shown) and did not produce tumors in vivo (Figure 4A). To determine the level of immunity induced, all tumor-free mice were challenged after 3 weeks with untreated B16 cells. The TSA-treated live plus apoptotic 50:50 mixture generated a significantly higher level of durable immunity (Figure 4B, 88% tumor-free) compared to the TSA-treated apoptotic inoculums (30% tumor-free) and H_2_O_2_-treated apoptotic cells failed to elicit long-term immunity. The lasting immunity induced by TSA-treated apoptotic cells is statistically significant compared to H_2_O_2_-treated apoptotic cells and untreated controls (p <0.04). These data suggest that TSA-treated apoptotic cells may play a role in immunity and TSA-treated preparations containing ~50% apoptotic cells, in addition to non-apoptotic cells, are most effective in inducing long-term immunity. Importantly, these data also suggest that generation of durable immunity by annexinV positive tumor cells may vary depending on the apoptosis-inducing agents [28].

### Treatment with epigenetically altered melanoma vaccines suppresses growing tumors

**Figure 5:**
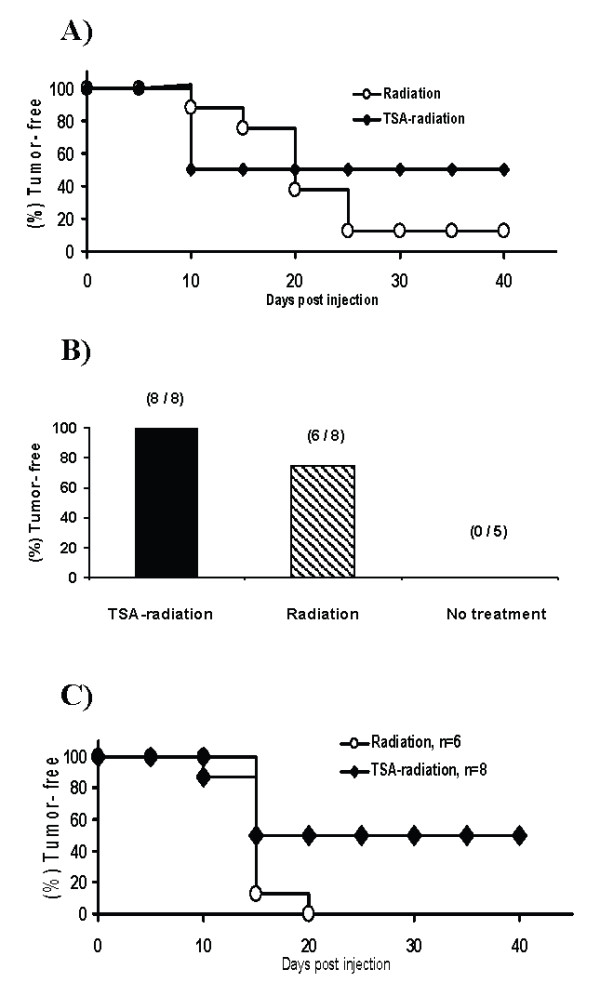
Prevention and treatment of melanoma by TSA-treated (irradiated) tumor cell vaccine. **A**) Vaccination with TSA-treated (500 nM for 48 h) and irradiated (2000 Gy) B16 cells prevents B16 tumor generation. Three weeks after s.c. inoculation of TSA-treated [TSA-radiation] or untreated [radiation] B16 (1 × 10^5^) cells in B6 mice (16 in each group); all tumor-free mice were challenged s.c. with wild type B16 (1 × 10^5^) cells and observed for 40 days. Percentage of tumor-free mice is presented in the Kaplan-Meier plot. **B**) Suppression of primary tumor growth by TSA-treated and irradiated melanoma vaccine. Groups of B6 mice bearing palpable B16 (5 days tumor growth) were treated s.c. with TSA-treated and irradiated B16 [TSA-radiation] or untreated irradiated B16 (1 × 10^5 ^cells) [radiation] in the opposite side of the trunk. A group of tumor-bearing mice did not receive any treatment [no treatment]. The number of tumor-free mice compared to total numbers used in each group is shown in parentheses at 42 days after treatment. **C**) Induction of long-term immunity in tumor-bearing mice treated with epigenetically altered melanoma vaccine. B16 tumor-bearing mice that became tumor-free following vaccine treatment were re-challenged s.c. with wild type B16 (1 × 10^5 ^cells). Kaplan-Meier plot shows tumor-free survival of mice in both TSA-radiation and radiation groups after re-challenge.

To evaluate the potential of clinical translation of the TSA-treated vaccine model, two sets of tumorigenesis experiments were performed. Firstly, to eliminate tumor growth from the vaccine inocula, we lethally irradiated tumor cells before inoculation and found that 100% of the mice injected with TSA-treated or untreated B16 cells (irradiated) did not develop tumor (data not shown). After 3 weeks, tumor-free mice from both groups were challenged with wild type B16; 50% of the TSA-radiation group and only 12.5% of the control-radiation group showed durable immunity (Figure 5A). These data suggest that killing the TSA-treated tumor cells with radiation does not reduce the vaccine efficacy. Additionally, to determine the efficacy of the irradiated vaccine in a therapeutic model, palpable B16 tumor-bearing mice were inoculated with TSA-treated or untreated B16 cells (irradiated) on the opposite side. As shown in Figure 5B and 5C, 100% of mice receiving a single injection of TSA-treated (irradiated) B16 vaccine were tumor-free at 42 days and 50% of them remained tumor-free 40 days after wild type tumor challenge. In 75% of tumor-bearing mice, the tumor regressed after injection with irradiated B16 cells (Figure 5B), but none of them were protected against challenge with wild type B16 (Figure 5C). These data demonstrate that vaccination with TSA-treated (irradiated) tumor cells can suppress primary tumor growth as well as promote long-term immunity in this treatment model.

## Discussion

This study demonstrates that vaccination with TSA-treated melanoma cells expressing both MHC class II and costimulatory molecules can generate tumor-specific durable immunity and that the vaccine is effective against an existing B16 tumor in a treatment protocol. The induction of tumor immunity described here requires CD8^+^, CD4^+ ^T and NK cells. Experiments performed in MHC class I knockouts and CD8^+ ^T cell antibody-depleted mice substantially reduced the anti-tumor effects of the vaccine thereby supporting the essential role of CD8^+ ^T cells in this model. Additionally, the partial abrogation of the immune responses in class II knockout mice, the lack of immunity in MHC double knockout mice and the tumorigenesis data in CD4^+ ^T cell antibody-depleted animals suggest that CD4^+ ^T cells are also essential in the generation of immunity. The data from T cell deficient or depleted mice together with the findings of cytotoxic and IFN-γ-secreting lymphocytes in immune mice in association with long term immunity demonstrate that a single dose of the TSA-treated tumor cell vaccine is capable of activating naïve T cells to generate cytotoxic effector and memory T cells. Our results also showed that depletion of NK cells completely abrogated immunity generation by TSA-treated B16 cells suggesting that NK cells, together with T cells, play an important role in initiation of immunity following epigenetic vaccination.

The association of immunity with enhanced class II and costimulatory molecule expression and in vitro activation of antigen-specific CD4^+ ^T cells by TSA-treated melanoma cells is consistent with the hypothesis of direct antigen presentation by tumor cells in vivo. That direct presentation could be a component of tumor immunity is suggested by: 1) the reported demonstration of direct presentation in viral and other tumor model systems [7,29]; 2) the effectiveness of tumor vaccines established by transfecting tumor cells with MHC and costimulatory genes [30]; and 3) the conversion of tumor cells to APCs by epigenetic agents discussed above and previously reported [14,18]. The substantial effectiveness of the TSA-modified tumor cell vaccine shown here, together with the delayed tumor generation data in MHC deficient chimeras, suggest a short-lived in vivo effect from direct antigen presentation. Our view is that cross-presentation is likely the major mechanism of T cell activation in most tumors (as outlined by others and reviewed in [2]) but that, in certain tumors and conditions, a component of direct presentation may exist. The important issue is whether clinically significant direct presentation can be established by appropriate treatments designed to convert a sizable portion of the tumor cells in the tumor bed to APCs. Since tumors may have a billion or more cells when first detected [31], converting even a small percentage of cells could potentially have significant effects. Furthermore, we would expect that Treg cells and other immunosuppressive mechanisms might also repress direct presentation. It remains to be determined whether combinations of the epigenetic vaccine with other modalities, such as adjuvants or anti-Treg antibody, can enhance the efficacy of direct antigen presentation by tumors. Current evidence suggests that sustained antigen presentation is important in promoting immunity and that the duration of antigen presentation is a major factor in activation of memory T cells [32,33]. The short-term effects seen in vivo in the direct presentation model could reflect the lack of sustained antigen presentation. Further experiments are required that include multiple injections, adjuvants with sustained antigen release, further adjustments of amount and concentration of the apoptotic population and other procedures employed for DC vaccines directed at enhancing and maintaining MHC/peptide complex and maintaining the adjuvant environment and activation state of the tumor [33,34].

Since HDACi affect the expression of many genes [12], we consider it likely that additional mechanisms other than direct presentation may contribute to epigenetically induced tumor immunity. In this regard, TSA treatment can enhance the expression of NKG2D ligands [35] on tumor cells, which may activate NK cell mediated immune responses and synergize with CD28 in inducing proliferation and IFN-γ production by CD8^+ ^T cells [36]. However, whether TSA induction of NKG2D ligands is mediated by chromatin or stress effects on tumor cells is uncertain. In addition, although our data and other reports suggest that NK cells can mediate immunity against tumor cells expressing NKG2D ligand [37] or MHC class I [38], the role of NK cells in generating a long-lasting memory immune response after epigenetic vaccination is uncertain. One possibility suggested by the strict requirement for NK cells is that NK cells interact with tumor cells treated with TSA (and expressing CD40) as described for DC [39,40] and enhance antigen presentation [33].

Apoptotic tumor cells could potentially contribute to immunity generation through several pathways. Previous studies suggested that phagocytosis of apoptotic cells by DCs in certain conditions can induce tolerance [41], while other reports demonstrate that cross-presentation of antigen from apoptotic tumor cells can generate tumor specific immune responses [42]. The experiments performed with TSA-treated apoptotic B16 preparations demonstrated that apoptotic tumor cells are capable of eliciting long-term immunity. The induction of immunity by apoptotic tumor cells has been related to their ability to induce inflammation and to mature DC, in part owing to the presence of heat shock proteins in apoptotic cell preparations, especially those containing necrotic cells [1,43,44]. Since apoptotic cells were defined by annexinV binding in our study, the presence of an^+ ^necrotic cells is likely in TSA-treated apoptotic preparations. It has been reported that apoptotic tumor cells produced by treatment with TSA have different expression patterns of MHC class II and costimulatory molecules compared to apoptotic cells elicited by L-phenylalanine mustard, H_2_O_2 _and γ-irradiation [28]. Moreover the data presented here shows that apoptotic tumor cell preparations produced by TSA, but not H_2_O_2_, treatment generated long-term immunity. The failure of the H_2_O_2_-treated apoptotic B16 cells to enhance immunity demonstrates a selective effect of TSA-treated apoptotic cells and suggests that apoptosis inducing agents should be carefully evaluated for differential effects in anti-cancer therapy. In this regard, recent evidence suggests that apoptotic cell death due to caspase-3 activation results in the release of bioactive lipid molecules, which promote DC maturation and pro-inflammatory responses [45]. Since TSA may induce apoptosis in certain tumor cells through a caspase-3 and mitochondria dependent pathway [46], it is possible that TSA-treated apoptotic B16 cells release bioactive lipids, which promote innate, perhaps TLR and NK mediated, immunity as well as adaptive responses.

The reported enhancement of DC vaccine potency by the addition of apoptosis promoting agents suggests a role for apoptotic cells in synergizing with live cells in eliciting immunity [47]. Our results showed that addition of TSA-treated non-apoptotic cells to apoptotic (50% an^- ^+ 50% an^+^) B16 inocula significantly improved the vaccine efficacy. Therefore, in addition to the conversion of tumor cells to APCs, the combination of replicating tumor cells producing high levels of antigen with an apoptotic adjuvant-like effect could explain the superiority of the high dose (500 nM) TSA-treated B16 vaccines compared with lower doses of TSA and the H_2_O_2_-treated cells. Also, since TSA alters chromatin and indirectly inhibits DNA methylation [48], it could potentially activate repressed endogenous tumor antigens, such as MAGE [49] and HMW-MAA [50] that may serve as epigenetically induced tumor antigens. Finally, HDACi modulates DNA damage (ATM/ATR) [35] and MAPK pathways [51] and could therefore serve as an intracellular adjuvant, which have been shown to enhance DC vaccine potential [34].

The data presented here suggests that an autologus tumor cell vaccine generated by HDACi in vitro is effective in both preventative and treatment models. Further work, currently in progress, will focus on obtaining more effective vaccines using different HDACi preparations, combination with adjuvant or anti-Treg as well as multiple vaccinations. We would envision future clinical protocols using this vaccine, most likely in combination with surgery and/or other strategies, to treat recurrence and metastasis of tumors. Clearly there are challenges and potential problems in adapting a cell-based vaccine of this type to the treatment of human cancer. If clinically effective vaccines could be developed, they would have the advantage of being histocompatible with the host and applicable without prior knowledge of the tumor antigens involved. These studies also suggest that current therapies with epigenetic agents could affect host immunity to the tumor and attention should be given to this aspect in ongoing and future clinical protocols using these agents systemically.

## Competing interests

The author(s) declare that they have no competing interests.

## Authors' contributions

A. Nazmul H. Khan – had the primary responsibility for designing and conducting all the experiments, data analysis and initial drafting of the manuscript; William J. Magner – was involved in study design, drafting and review of the manuscript; Thomas B. Tomasi – conceived the study, contributed to the design and subsequent drafts of the manuscript. All authors read and approved the final manuscript.

## Supplementary Material

Additional File 1 Characterization of lymphocyte re-constitution in bone marrow chimeras. The data provided characterize lymphocyte reconstitution in the chimeras by flow cytometric analysis of peripheral blood lymphocytes isolated from chimeras. Click here for file

## References

[B1] Restifo NP (2000). Building better vaccines: how apoptotic cell death can induce inflammation and activate innate and adaptive immunity. Curr Opin Immunol.

[B2] Heath WR, Carbone FR (2001). Cross-presentation, dendritic cells, tolerance and immunity. Annu Rev Immunol.

[B3] Nowak AK, Lake RA, Marzo AL, Scott B, Heath WR, Collins EJ, Frelinger JA, Robinson BW (2003). Induction of tumor cell apoptosis in vivo increases tumor antigen cross-presentation, cross-priming rather than cross-tolerizing host tumor-specific CD8 T cells. J Immunol.

[B4] Khan AN, Magner WJ, Tomasi TB (2004). An epigenetically altered tumor cell vaccine. Cancer Immunol Immunother.

[B5] Qi L, Rojas JM, Ostrand-Rosenberg S (2000). Tumor cells present MHC class II-restricted nuclear and mitochondrial antigens and are the predominant antigen presenting cells in vivo. J Immunol.

[B6] Knutson KL, Disis ML (2005). Tumor antigen-specific T helper cells in cancer immunity and immunotherapy. Cancer Immunol Immunother.

[B7] Schoenberger SP, Jonges LE, Mooijaart RJ, Hartgers F, Toes RE, Kast WM, Melief CJ, Offringa R (1998). Efficient direct priming of tumor-specific cytotoxic T lymphocyte in vivo by an engineered APC. Cancer Res.

[B8] Pulaski BA, Ostrand-Rosenberg S (1998). Reduction of established spontaneous mammary carcinoma metastases following immunotherapy with major histocompatibility complex class II and B7.1 cell-based tumor vaccines. Cancer Res.

[B9] Mortara L, Castellani P, Meazza R, Tosi G, De Lerma Barbaro A, Procopio FA, Comes A, Zardi L, Ferrini S, Accolla RS (2006). CIITA-induced MHC class II expression in mammary adenocarcinoma leads to a Th1 polarization of the tumor microenvironment, tumor rejection, and specific antitumor memory. Clin Cancer Res.

[B10] Jabrane-Ferrat N, Campbell MJ, Esserman LJ, Peterlin BM (2006). Challenge with mammary tumor cells expressing MHC class II and CD80 prevents the development of spontaneously arising tumors in MMTV-neu transgenic mice. Cancer Gene Ther.

[B11] Marincola FM, Jaffee EM, Hicklin DJ, Ferrone S (2000). Escape of human solid tumors from T-cell recognition: molecular mechanisms and functional significance. Adv Immunol.

[B12] Tomasi TB, Magner WJ, Khan AN (2006). Epigenetic regulation of immune escape genes in cancer. Cancer Immunol Immunother.

[B13] Magner WJ, Kazim AL, Stewart C, Romano MA, Catalano G, Grande C, Keiser N, Santaniello F, Tomasi TB (2000). Activation of MHC class I, II, and CD40 gene expression by histone deacetylase inhibitors. J Immunol.

[B14] Khan AN, Gregorie CJ, Tomasi TB A role for chromatin in the regulation of TAP, LMP, Tapasin genes and MHC class I antigen presentation. Cancer Immunol Immunother.

[B15] Jenuwein T, Allis CD (2001). Translating the histone code. Science.

[B16] Mariadason JM, Corner GA, Augenlicht LH (2000). Genetic reprogramming in pathways of colonic cell maturation induced by short chain fatty acids: comparison with trichostatin A, sulindac, and curcumin and implications for chemoprevention of colon cancer. Cancer Res.

[B17] Marks PA, Richon VM, Breslow R, Rifkind RA (2001). Histone deacetylase inhibitors as new cancer drugs. Curr Opin Oncol.

[B18] Chou SD, Khan AN, Magner WJ, Tomasi TB (2005). Histone acetylation regulates the cell type specific CIITA promoters, MHC class II expression and antigen presentation in tumor cells. Int Immunol.

[B19] Manjili MH, Wang XY, Chen X, Martin T, Repasky EA, Henderson R, Subjeck JR (2003). HSP110-HER2/neu chaperone complex vaccine induces protective immunity against spontaneous mammary tumors in HER-2/neu transgenic mice. J Immunol.

[B20] Sotomayor EM, Borrello I, Rattis FM, Cuenca AG, Abrams J, Staveley-O'Carroll K, Levitsky HI (2001). Cross-presentation of tumor antigens by bone marrow-derived antigen-presenting cells is the dominant mechanism in the induction of T-cell tolerance during B-cell lymphoma progression. Blood.

[B21] Lenz LL, Butz EA, Bevan MJ (2000). Requirements for bone marrow-derived antigen-presenting cells in priming cytotoxic T cell responses to intracellular pathogens. J Exp Med.

[B22] Nonacs RM, Witmer-Pack MD, Steinman RM (1992). Enrichment of dendritic cells by plastic adherence and EA Rosetting. Current Protocols in Immunology.

[B23] Grusby MJ, Glimcher LH (1995). Immune responses in MHC class II-deficient mice. Annu Rev Immunol.

[B24] Degli-Esposti MA, Smyth MJ (2005). Close encounters of different kinds: dendritic cells and NK cells take centre stage. Nat Rev Immunol.

[B25] Hamerman JA, Ogasawara K, Lanier LL (2005). NK cells in innate immunity. Curr Opin Immunol.

[B26] Vallera DA, Soderling CC, Orosz CG (1985). Assessment of immunocompetence by limiting dilution analysis in long-term T cell depletion chimeras transplanted across the MHC barrier. Transplantation.

[B27] Hargadon KM, Brinkman CC, Sheasley-O'neill SL, Nichols LA, Bullock TN, Engelhard VH (2006). Incomplete differentiation of antigen-specific CD8 T cells in tumor-draining lymph nodes. J Immunol.

[B28] Magner WJ, Tomasi TB (2005). Apoptotic and necrotic cells induced by different agents vary in their expression of MHC and costimulatory genes. Mol Immunol.

[B29] Brady MS, Eckels DD, Ree SY, Schultheiss KE, Lee JS (1996). MHC class II-mediated antigen presentation by melanoma cells. J Immunother Emphasis Tumor Immunol.

[B30] Ostrand-Rosenberg S, Pulaski BA, Clements VK, Qi L, Pipeling MR, Hanyok LA (1999). Cell-based vaccines for the stimulation of immunity to metastatic cancers. Immunol Rev.

[B31] Friberg S, Mattson S (1997). On the growth rates of human malignant tumors: implications for medical decision making. J Surg Oncol.

[B32] Iezzi G, Karjalainen K, Lanzavecchia A (1998). The duration of antigenic stimulation determines the fate of naive and effector T cells. Immunity.

[B33] Obst R, van Santen HM, Melamed R, Kamphorst AO, Benoist C, Mathis D (2007). Sustained antigen presentation can promote an immunogenic T cell response, like dendritic cell activation. Proc Natl Acad Sci USA.

[B34] Andreakos E, Williams RO, Wales J, Foxwell BM, Feldmann M (2006). Activation of NF-kappaB by the intracellular expression of NF-kappaB-inducing kinase acts as a powerful vaccine adjuvant. Proc Natl Acad Sci USA.

[B35] Gasser S, Orsulic S, Brown EJ, Raulet DH (2005). The DNA damage pathway regulates innate immune system ligands of the NKG2D receptor. Nature.

[B36] Ehrlich LI, Ogasawara K, Hamerman JA, Takaki R, Zingoni A, Allison JP, Lanier LL (2005). Engagement of NKG2D by cognate ligand or antibody alone is insufficient to mediate costimulation of human and mouse CD8+ T cells. J Immunol.

[B37] Zhou H, Luo Y, Lo JF, Kaplan CD, Mizutani M, Mizutani N, Lee JD, Primus FJ, Becker JC, Xiang R (2005). DNA-based vaccines activate innate and adaptive antitumor immunity by engaging the NKG2D receptor. Proc Natl Acad Sci USA.

[B38] Kamiryo Y, Yajima T, Saito K, Nishimura H, Fushimi T, Ohshima Y, Tsukamoto Y, Naito S, Yoshikai Y (2005). Soluble branched (1,4)-beta-D-glucans from Acetobacter species enhance antitumor activities against MHC class I-negative and -positive malignant melanoma through augmented NK activity and cytotoxic T-cell response. Int J Cancer.

[B39] Walzer T, Dalod M, Robbins SH, Zitvogel L, Vivier E (2005). Natural-killer cells and dendritic cells: "l'union fait la force". Blood.

[B40] Munz C, Steinman RM, Fujii S (2005). Dendritic cell maturation by innate lymphocytes: coordinated stimulation of innate and adaptive immunity. J Exp Med.

[B41] Steinman RM, Turley S, Mellman I, Inaba K (2000). The induction of tolerance by dendritic cells that have captured apoptotic cells. J Exp Med.

[B42] Nouri-Shirazi M, Banchereau J, Bell D, Burkeholder S, Kraus ET, Davoust J, Palucka KA (2000). Dendritic cells capture killed tumor cells and present their antigens to elicit tumor-specific immune responses. J Immunol.

[B43] Basu S, Binder RJ, Suto R, Anderson KM, Srivastava PK (2000). Necrotic but not apoptotic cell death releases heat shock proteins, which deliver a partial maturation signal to dendritic cells and activate the NF-kappa B pathway. Int Immunol.

[B44] Inaba K, Turley S, Yamaide F, Iyoda T, Mahnke K, Inaba M, Pack M, Subklewe M, Sauter B, Sheff D (1998). Efficient presentation of phagocytosed cellular fragments on the major histocompatibility complex class II products of dendritic cells. J Exp Med.

[B45] Albert ML (2004). Death-defying immunity: do apoptotic cells influence antigen processing and presentation?. Nat Rev Immunol.

[B46] Roh MS, Kim CW, Park BS, Kim GC, Jeong JH, Kwon HC, Suh DJ, Cho KH, Yee SB, Yoo YH (2004). Mechanism of histone deacetylase inhibitor Trichostatin A induced apoptosis in human osteosarcoma cells. Apoptosis.

[B47] Candido KA, Shimizu K, McLaughlin JC, Kunkel R, Fuller JA, Redman BG, Thomas EK, Nickoloff BJ, Mule JJ (2001). Local administration of dendritic cells inhibits established breast tumor growth: implications for apoptosis-inducing agents. Cancer Res.

[B48] Bestor TH (1998). Gene silencing. Methylation meets acetylation. Nature.

[B49] Wischnewski F, Pantel K, Schwarzenbach H (2006). Promoter demethylation and histone acetylation mediate gene expression of MAGE-A1, -A2, -A3, and -A12 in human cancer cells. Mol Cancer Res.

[B50] Luo W, Wang X, Kageshita T, Wakasugi S, Karpf AR, Ferrone S (2006). Regulation of high molecular weight-melanoma associated antigen (HMW-MAA) gene expression by promoter DNA methylation in human melanoma cells. Oncogene.

[B51] Yu C, Friday BB, Lai JP, McCollum A, Atadja P, Roberts LR, Adjei AA (2007). Abrogation of MAPK and Akt signaling by AEE788 synergistically potentiates histone deacetylase inhibitor-induced apoptosis through reactive oxygen species generation. Clin Cancer Res.

