# Supplementary material for: An epigenetic vaccine model active in the prevention and treatment of melanoma
Source: J Transl Med. 2007 Dec 10;5:64. doi: 10.1186/1479-5876-5-64 (PMC2231344; doi:10.1186/1479-5876-5-64)
Supplement: Additional File 1 — Characterization of lymphocyte re-constitution in bone marrow chimeras. The data provided characterize lymphocyte reconstitution in the chimeras by flow cytometric analysis of peripheral blood lymphocytes isolated from chimeras. [file 1479-5876-5-64-S1.pdf]

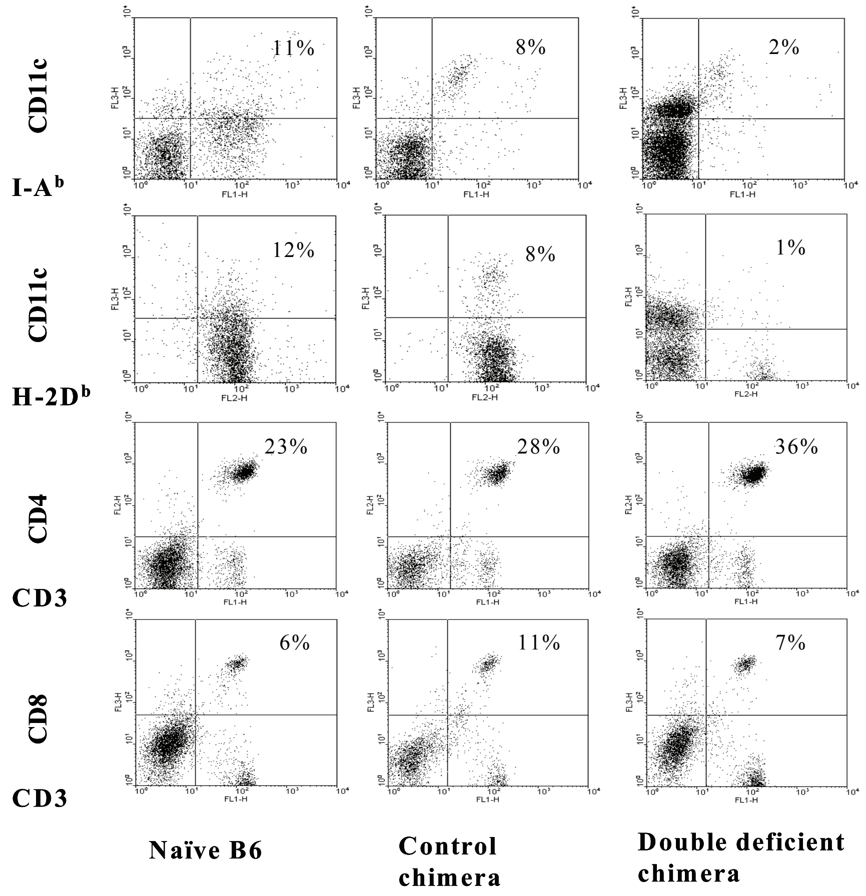


Additional figure 1: Presence of T cells in bone marrow chimeras and reduced percentage of MHC expressing APCs in MHC double deficient chimeras. MHC class I and class II deficient and control chimeric mice were produced by transplantation of BM from *class* $I^{-/-}$-$II^{-/-}$ or B6 donors into irradiated B6 recipients, respectively. PBLs isolated from control and double MHC deficient chimeras, 6 weeks after transplantation, were analyzed by flow cytometry for I-A$^b$ and H-2D$^b$ expression in CD11c$^+$ APCs and for CD4 and CD8 expression in CD3$^+$ T cells. PBLs isolated from naïve mice were used as controls. The percentage of MHC expressing APCs, CD4$^+$ and CD8$^+$ T cells are indicated in the appropriate quadrant of their respective plots.
